# In vitro evaluation of the tension band suture method for proximal humerus fracture treatment

**DOI:** 10.1186/s13018-020-01890-5

**Published:** 2020-08-20

**Authors:** Hideaki Ishii, Takanori Shintaku, Shu Yoshizawa, Misato Sakamoto, Takao Kaneko, Yoshiro Musha, Hiroyasu Ikegami

**Affiliations:** Department of Orthopaedic Surgery (Ohashi), School of Medicine, Toho University, 2-22-36 Ohashi, Meguro-ku, Tokyo, 153-8515 Japan

**Keywords:** Tension band suture, Washer, Proximal humeral fractures, FiberWire, Ethibond, Surgilon, Targon PH-P, Intramedullary nail

## Abstract

**Background:**

Proximal humeral fractures are common, and more than half occur in patients over 65 years of age. Operative treatment may be recommended for displaced, complicated fractures; however, surgery may lead to displacement of the greater tuberosity or humeral head. Supplemental tension band sutures have been recommended to prevent such a complication. In this study, we investigate the best combination of suture, washer, and threading angle for proximal humeral fractures from a mechanical view.

**Methods:**

The mechanical durability of 18 combinations of suture materials (Fiberwire, Ethibond, and Surgilon), threading washers (ring washer, disc washer), and threading angles (15 or 45°) were examined via a cyclic loading test.

**Results:**

The most durable combination in the cyclic loading test consisted of threading the Fiber Wire to the washer ring using only one hole (ring washer-1) at 45°. In contrast, the most vulnerable combination was threading Ethibond to the washer disc at 15°. Breakage of all suture materials occurred at the suture-washer interface, and no failure or loosening of the knots was observed. FiberWire gradually eroded until the loss of equilibrium; whereas the rupture of Ethibond and Surgilon occurred suddenly.

**Conclusions:**

From a mechanical viewpoint, we demonstrated that applying a supplemental tension band suture using FiberWire with a single-hole ring washer threaded at a wider angle is recommended.

## Background

Proximal humeral fractures account for 5.7% of all fractures, and incidence increases with age [1]. They are the third most frequent fracture in the elderly, after fractures of the hip and distal radius fractures, and more than half occur in patients over 65 years of age. Low bone mineral density and a high fall risk score are known risk factors [2, 3]. Non-operative treatment is reasonable for most stable, minimally displaced proximal humeral fractures [4]. However, approximately 20% of displaced, complicated fractures may benefit from operative treatment [5]. Many surgical techniques have been described to prevent complications, such as humeral malunions, non-unions, stiffness, and post-traumatic arthrosis, which can be significantly disabling [6]. Open reduction and internal fixation (ORIF), using either angular or sliding stable antegrade locking intramedullary nails (IMN) or anatomically designed proximal humeral angular stable plates, is one standard surgical treatment option for proximal humeral fractures [7]. These modern implants offer high primary stability, which can allow early functional exercises and provide good to excellent results in the majority of patients, with an acceptable complication rate [8, 9]. However, considerable issues exist after surgery, especially for the elderly due to their poor bone quality, and no single approach is considered to be the standard of care [5]. Displacement of the greater tuberosity and humeral head are common complications of surgery and can lead to malunion or nonunion [7, 10, 11]. Several studies have recommended tension band sutures for the prevention of this complication [12–17]. However, to the best of our knowledge, no study has focused on the variety and combination of components, such as the washer, suture material, and threading angle, of tension band sutures in detail. The purpose of this study is to explore the most suitable method of tension band suture to treat proximal humeral fractures using IMN.

## Methods

### Experimental device

**Fig. 1:**
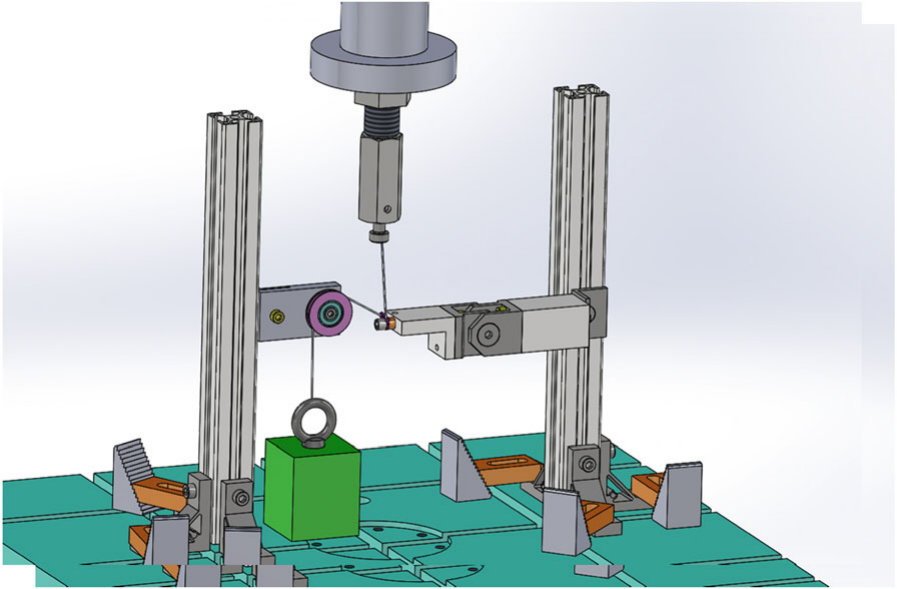
Schema of Servo pulsar used for loading test. Suture materials were passed through the washer which was tightened with screws. The edge of thread was tied to rod and weight rigidly by use of 5 square knots

**Fig. 2:**
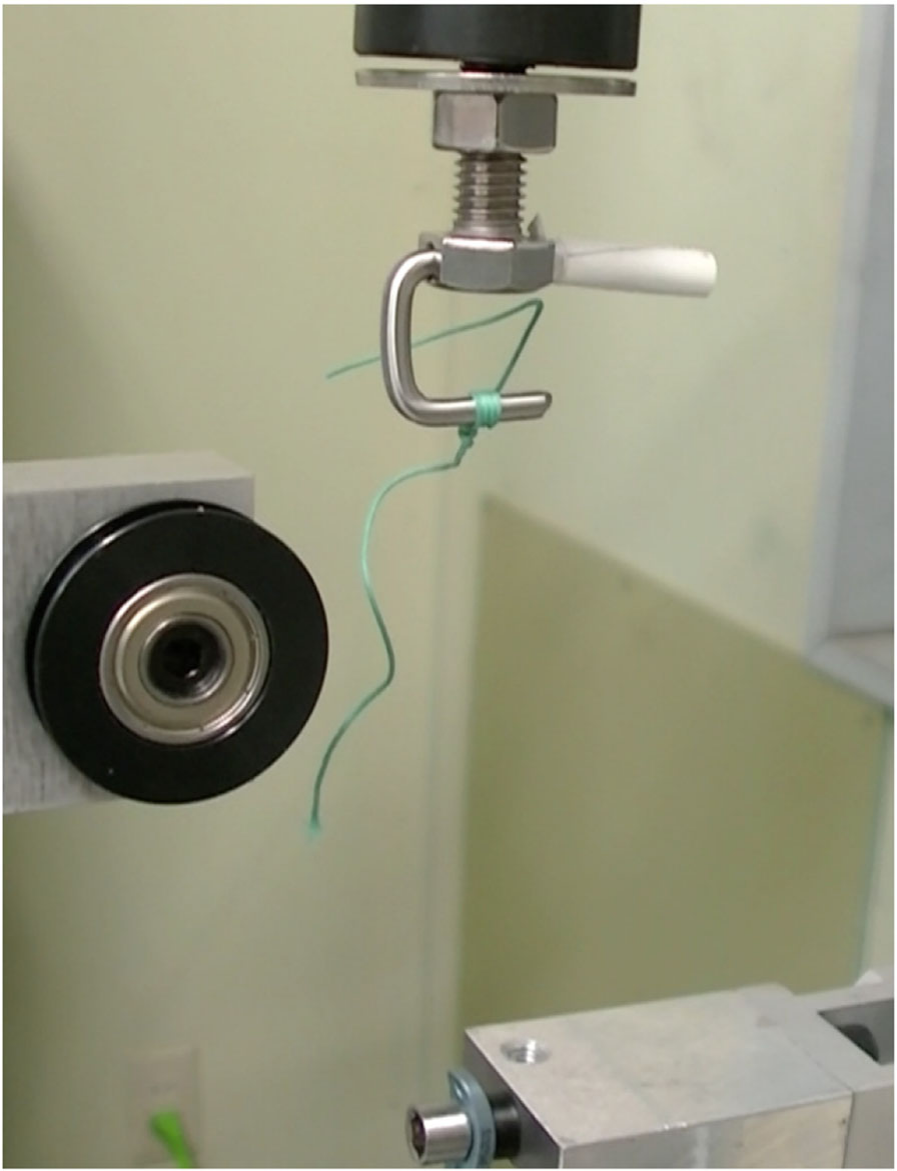
A broken suture

The testing device consisted of a fixed washer, the suture material, and a weight. Non-absorbable sutures were passed through the washers, tightened with screws vertically to the long axis, and connected to a weight (Fig. 1). The weight was set to 5 kgf, in accordance with an advanced trial. A cyclic loading test was performed with reciprocating motion at 0.2 Hz using a Servo pulsar (EHF-LV005k1-A10; Shimadzu Corp., Kyoto, Japan) in 10 mm strokes. The load ranged from approximately 15 N to 90 N, changing every second in conjunction with the motion of the piston. The maximal load of 90 N was considered to be well within the physiologic range, representing only 30% of the load that could be delivered by the maximal contraction of muscles [18]. The loading cycle was continued until failure of the suture material (Fig. 2) and observation of the weight touching the ground. The number of vertical motions was counted. The mechanical durability of 18 combinations of suture materials (*n* = 3), threading washers (*n* = 3), and threading angles (*n* = 2) were compared. Each combination was tested in the same condition three times.

### Suture materials

Three different non-absorbable suture materials commonly used in orthopedic surgery were compared: FiberWire (Arthrex, Inc., FL, USA); Ethibond (ETHICON, Inc., NJ, USA); and Surgilon (Medtronic, Inc., MN, USA). All sutures used were of standard size (no. 2) and kept moist throughout testing with 0.9% of saline solution to mimic the internal environment. One end of each suture was tied to the rod, and the other side was tied to the weight after threading the washer with five square knots.

### Washers and threading methods

**Fig. 3:**
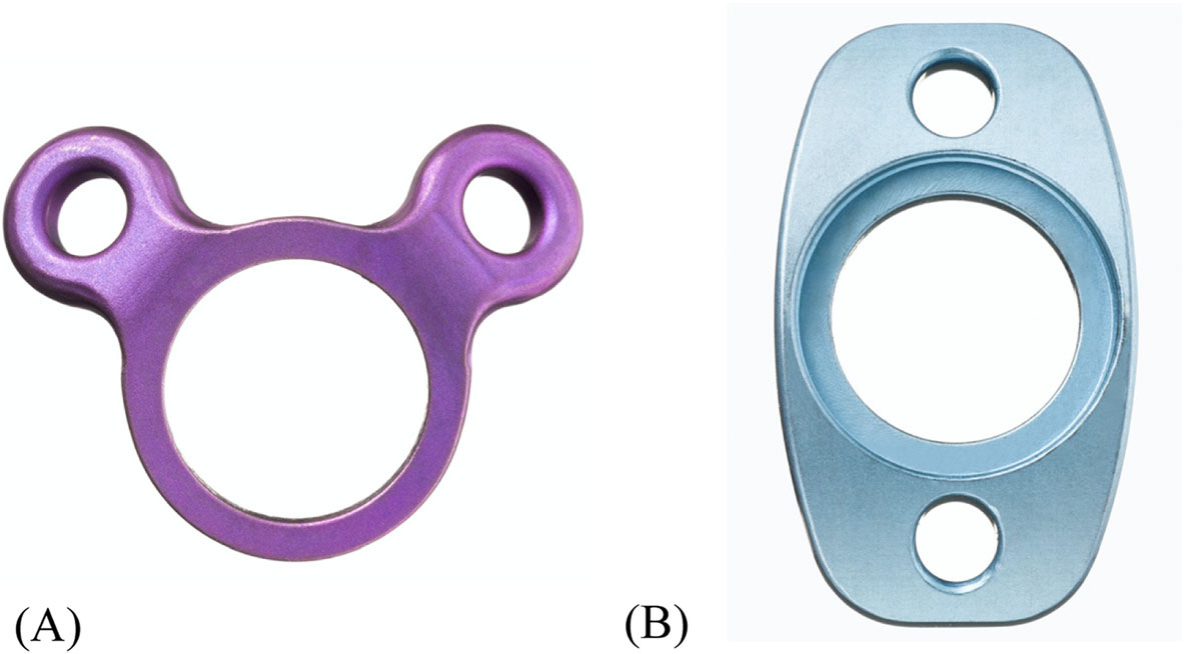
Two washers used for tension band suture in this study. Ring washer (**a**) and disc washer (**b**) are attached to Targon PH-P

**Fig. 4:**
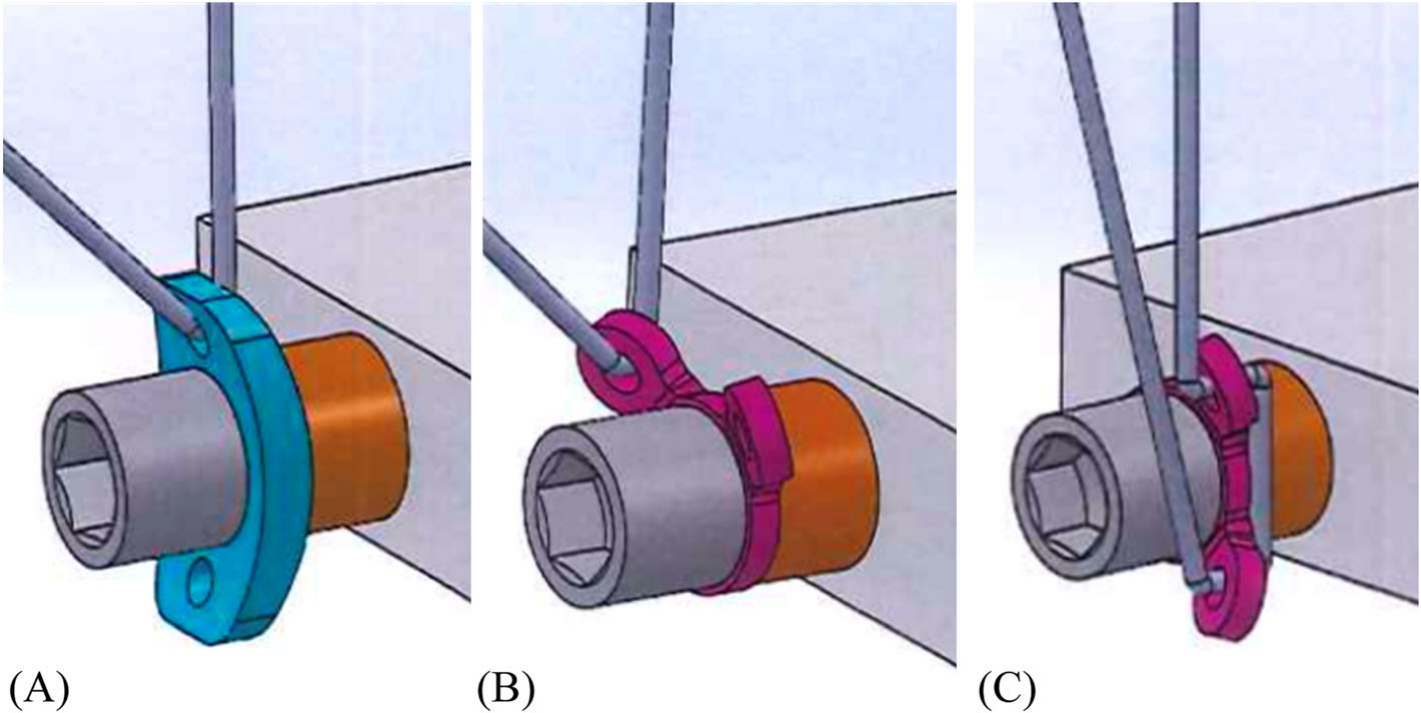
Three threading methods are shown. Using (**a**) and ring washer. Threading a hole to ring washer was defined ring washer-1 (**b**) and two holes ring washer-2 (**c**)

**Fig. 5:**
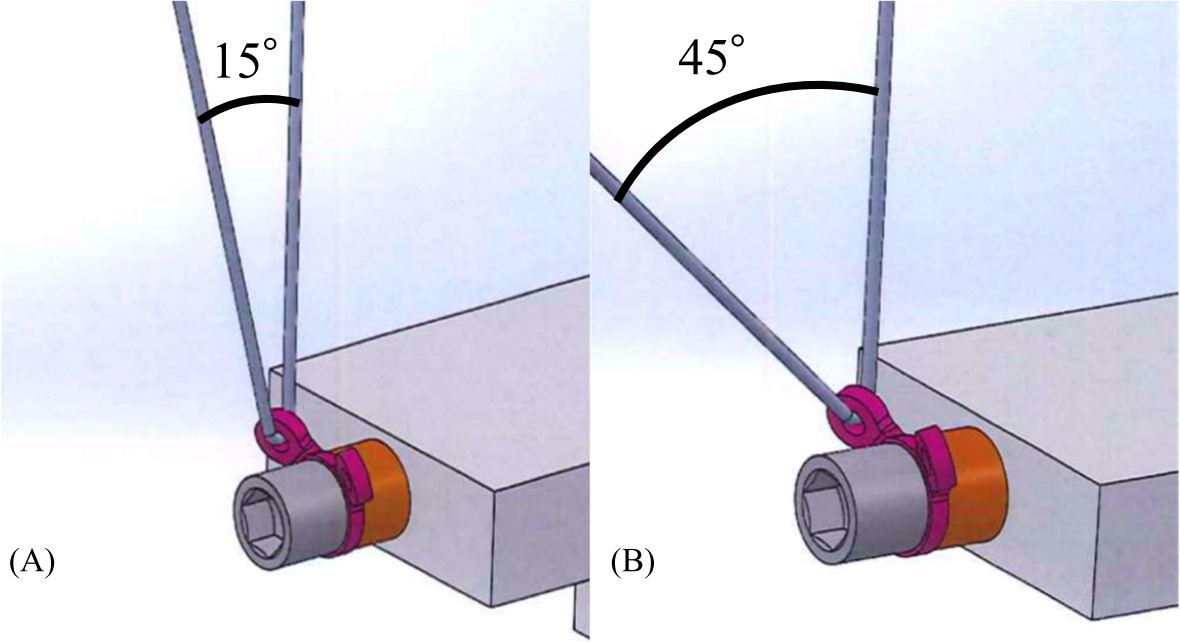
Suture materials were thread to washer by 15° (**a**) or 45° (**b**)

Two different TARGON PH-P (AESCULAP, Inc., PA, USA) washers (ring washer, disc washer) were utilized (Fig. 3). The ring washer is pure titanium and has two circular suture holes at the top, adjoining on the same side. The disc washer is made of a titanium alloy, consisting of 90% titanium, 6% aluminum, and 4% vanadium, and suture holes are located on both sides of the washer. The suture holes for each washer were 1.8 mm in diameter. The three threading procedures used with these washers are shown in Fig. 4. To distinguish different threading methods using the same washer, threading only one hole of the ring washer was denominated “ring washer-1” and two holes “ring washer-2.” When locating the reference point of the washer-suture interface, the angle of the thread was set to 15 or 45° (Fig. 5).

### Statistical analysis

An analysis of qualification typeIwas performed to elucidate the most impactful element for a substantial tension band suture. The Kruskal-Wallis test was used to compare different suture materials and threading methods. The Bonferroni procedure was conducted for post hoc comparisons to clarify groups when statistical significance was detected. The effect of the threading angle was tested by the Mann-Whitney *U* test. All statistical analyses were performed using IBM SPSS Statistics24 (IBM Japan, Tokyo, Japan) software. A *P* < 0.05 was considered significant.

## Results

The results of each combination are shown in Table 1. Larger numeric values indicate resistance to frictional force and loading. The most durable combination in the cyclic loading test was threading the FiberWire to the washer ring using only one hole (ring washer-1) at 45°, which averaged 173 vertical motions before thread rupture. In contrast, the most vulnerable combination was threading Ethibond to the disc washer at 15°, averaging only 1.3 vertical motions. More than a hundredfold difference in durability existed between these two combinations. According to the analysis of qualification typeI, FiberWire was overwhelmingly a center of interest by 59% of the contribution ratio, followed by the ring washer-1 at 21%, the disc washer at 10%, and 45° at 4%. Among these eight experimental elements, Ethibond and 15° had the most negative effect on the results. Hence, an additional analysis of angles and washers among FiberWire group was performed.

**Table 1:** Number of times to failure for each combination

	Ethibond			FiberWire			Surgilon		
15°	#1	#2	#3	#1	#2	#3	#1	#2	#3
Disc washer	1	2	1	46	60	58	2	2	2
Ring washer-1	2	2	2	137	126	122	3	3	3
Ring washer-2	4	2	3	22	20	73	3	4	5
45°	#1	#2	#3	#1	#2	#3	#1	#2	#3
Disc washer	2	2	1	172	182	63	3	2	2
Ring washer-1	2	2	2	219	157	143	3	2	3
Ring washer-2	4	3	4	17	36	20	5	4	5

### Suture materials

The FiberWire group, mean 92.9 (SD 64.6), demonstrated prominent strength in the average number of reciprocating motions, compared to the Ethibond, mean 2.3 (SD 0.9), and Surgilon groups, mean 3.1 (SD 1.0) (*P* < 0.001) (Table 2). Breakage of all suture materials occurred at the suture-washer interface, and no failure or loosening of the knots was observed. The rupture pattern differed according to the suture material: FiberWire gradually eroded until the loss of equilibrium; whereas the rupture of Ethibond and Surgilon occurred suddenly.

**Table 2:** Average number of times to failure for each suture

Suture group	Number of reciprocating motion
FiberWire	92.9 (64.6)
Ethibond	2.3 (0.9)
Surgilon	3.1 (1.0)
*P*	< .001

Values are mean

Abbreviation: *SD* standard deviation

### Angle of suture

In comparing the two suture angles, the 45° group, mean 38.9 (SD 68.1), showed a higher average than the 15° group, mean 26.3 (SD 42.2); however, it was without significance (*P* = 0.57). The superiority of the wider angle also applied to each suture material, but again, the difference was not significant (Table 3).

**Table 3:** Average number of times to failure for each suture by different angles

Angles	FiberWire	Ethibond	Surgilon	Total
15°	73.8 (41.9)	2.1 (0.9)	3.0 (1.0)	26.3 (42.2)
45°	111.0 (74.7)	2.4 (1.0)	3.2 (1.1)	38.9 (68.1)
*P*	.29	.46	.74	.57

Values are mean

Abbreviation: *SD* standard deviation

### Washer and threading methods

Regarding the threading procedure, the ring washer-1 group had the highest endurance on average, mean 51.8 (SD 72.4), followed by the disc washer group, mean 33.5 (SD 55.6), and the ring washer-2 group, mean 13.0 (SD 17.2). Statistical significance was not observed among these three groups (*P* = 0.15); however, there was a significant difference in the threading methods of the FiberWire group (*P* = 0.006) (Table 4). The post hoc test revealed a significant difference in the durability of the ring washer-2, mean 31.3 (SD 21.5), and ring washer-1, mean 150.7 (SD 35.7), among FiberWire groups (*P* < 0.001).

**Table 4:** Average number of times to failure by threading methods

Threading methods	FiberWire	All sutures
Disc washer	96.8 (62.4)	33.5 (55.6)
Ring washer-1	150.7 (35.7)	51.8 (72.4)
Ring washer-2	31.3 (21.5)	13.0 (21.5)
*P*	.006	.15

Values are mean

Abbreviation: *SD* standard deviation

## Discussion

The multiple fixation techniques described in literature indicate that the optimal treatment for displaced proximal humeral fractures continues to be controversial [7, 8, 11, 19–21]. The preferred operation technique depends on fracture type, patient age, bone quality, and functional expectation. Surgery, using an angular and sliding stable antegrade nail (Targon PH), is a standard treatment option which can provide good functional results [10, 22, 23]. Supplemental tension band sutures are recommended for proximal humeral fracture treatment in a myriad of literature with favorable clinical results [12–17]. Badman et al. advocated that effectiveness derives from the counterforce to the natural deforming forces of the rotator cuff [12]. According to Park et al., tension band sutures placed between the rotator cuff and the head of the interlocking screw or washer, using no. 5 Ethibond suture material, increase the stability of the bone fragment with good postoperative shoulder function [15]. Badman et al. and Shukla et al. reported that locked plating with tension band rotator cuff fixation using a minimum of four or five no. 2 FiberWire sutures can prevent fixation failure and result in favorable clinical outcomes [12, 17]. Micic et al. emphasized the importance of applying a tension band suture over the tuberosity for additional stability; they report that negligence of this procedure is a risk factor for revision surgery [24]. On the other hand, there is a contradictory result which reports the invalidity of the tension band suture. Arvesen et al. performed a cadaveric study and concluded that tension-relieving rotator cuff sutures with no. 5 FiberWire do not add stability to the repair of 3-part proximal humeral fractures [25]. Furthermore, Voigt et al. also reported no contribution to reduce interfragmentary motion by additive fiber-cerclages in unstable 3-part fracture model with an intact rotator cuff [26]. The necessity of the tension band suture is yet controversial and heterogeneity of surgical indication exists. Moreover, suture materials, artifacts, and threading methods for tension band suture vary in the literature, which hinders discussions of its effectiveness. In this study, we attempted to present an ideal method from a mechanical viewpoint by focusing on the combination of three essential elements of the tension band suture: the suture material, threading angle, and washer.

FiberWire, a representative non-absorbable suture made of multi-strand, long-chain, ultra-high molecular weight polyethylene (UHMWPE), demonstrated higher strength than the other conventional sutures as Barber et al. reported; its superiority showed remarkable statistical significance in our study (*P* < 0.001) [27]. Moreover, the rupture pattern differed between FiberWire and the other suture materials. This might be ascribed to its structural composition and loading type. FiberWire consists of a UHMWPE core with a braided jacket of polyester and UHMWPE; whereas Ethibond and Surgilon are made of polyester and nylon with a braided structure coated with polybutylate and silicone. Wright et al. verified that FiberWire’s non-braided core, protected in its polyester jacket, resists elongation, and enables it to maintain strength, even when the suture is partially cut [28]. In most previous biomechanical experiments, the load to failure tensile tests is performed by mere continuous traction to the suture [18]. We performed cyclic loading in this study to replicate the type of load for which the tension band suture is considered to be exposed after surgery by the motion of the shoulder joint. Frictional force occurs repeatedly between the suture material and washer, in addition to tensile force. We think the gradual erosion of FiberWire by frictional force might be a consequence of the structural characteristics mentioned above. In spite of this distinctive property of FiberWire, Abbi et al. and Barber et al. reported that knot slippage occurred more frequently with FiberWire than Ethibond, which must be considered another mode of tension band suture failure [27, 29]. In our study, there was no knot slippage, regardless of suture materials, utilizing five square knots tied on each end.

Theoretically, when loading an identical tensile force to suture material, the normal force at the contact point between the washer and the suture material increases as the threaded suture makes an acute angle. As a result of this larger dynamic friction force, the 15° group had a tendency to be vulnerable; although no statistical significance existed between angle groups. We assume that threading the suture to the washer at a wider angle is desirable for rupture prevention. However, in clinical settings, the threading angle is affected by multiple conditions, such as design of artifact, bone fragments, and soft tissue.

Artifacts used for tension band sutures also play an important role. Generally, when treating with plates, dedicated eyelets in the plate are used to thread the suture [12, 17]. However, Cho et al. illustrated the difficulty in providing tension to sutures using eyelets in the plates because the knots might eventually loosen [13]. There are plural methods for tension band sutures using IMN. Hao et al. introduced a technique to augment tuberosity fixation by threading suture holes on the interlocking screws [30]. This might potentially have the same issue as threading to eyelets in the plate; additionally, the contraction of the rotator cuff can lead to screw backout. Park et al. performed tension band and locking sutures in addition to IMN and reported good clinical outcomes [16]. They hung the sutures only at the head of the interlocking screw, which we consider technically difficult with a potential risk of suture slippage or knot failure. To prevent these risks, washers were introduced. Cho et al. used two washers with plates to interpose the suture material and transmit the tension through the sutures [13]. Kim et al. employed a washer to secure the suture and compensate for the shortcomings of the tension band sutures with IMN [14]. We advocate this technique and are attempting to refine the method. When threading the suture to the washer, frictional force becomes a problem. The type of washer did not significantly affect the result when threading to a single hole; however, threading both holes of the washer ring in succession (washer ring-2) militated against the durability. Thus, engendering frictional wear at two points is a risk for early rupture.

Our study has several limitations. This is an in vitro study, so our model does not completely replicate the in vivo environment. Different external forces might act on the tension band suture when using a bone model of proximal humeral fracture. Additional cadaveric study might reveal those dynamics. Also, the sample size for each combination was limited to three, because we used a brand-new washer for each trial to ensure a uniform environment.

## Conclusion

Our study demonstrated that a supplemental tension band suture using FiberWire with a single-hole ring washer threaded at a wider angle is recommended from a mechanical viewpoint.

## Data Availability

All data generated or analyzed during this study are included in this published article.

## Abbreviations

ORIF: Open reduction and internal fixation
IMN: Intramedullary nails
UHMWPE: Ultra-high molecular weight polyethylene
